# Long-term exercise training and inflammatory biomarkers in healthy subjects: a meta-analysis of randomized controlled trials

**DOI:** 10.3389/fpsyg.2023.1253329

**Published:** 2023-08-30

**Authors:** Ya-Hai Wang, Jingwang Tan, Huan-Huan Zhou, Meng Cao, Yu Zou

**Affiliations:** ^1^College of Arts and Physical Education, Nanchang Normal College of Applied Technology, Nanchang, China; ^2^Department of Sport and Exercise Science, College of Education, Zhejiang University, Hangzhou, China; ^3^Department of Nutrition and Food Hygiene, Hubei Key Laboratory of Food Nutrition and Safety, Tongji Medical College, Huazhong University of Science and Technology, Wuhan, China; ^4^Department of Physical Education, College of Sport, Shenzhen University, Shenzhen, China

**Keywords:** long-term exercise training, inflammation, interleukin-6, C-reactive protein, tumor necrosis factor alpha, meta-analysis

## Abstract

**Objective:**

This meta-analysis investigated the effect of long-term exercise training (ET) including aerobic, resistance, and multicomponent ET on the levels of inflammatory biomarkers in randomized controlled trials (RCTs) involving healthy subjects.

**Methods:**

We searched seven databases for articles until May 1st, 2023. A random-effect meta-analysis, subgroup analysis, meta-regressions as well as trim and fill method were conducted using STATA 16.0.

**Result:**

Thirty-eight studies were included in the meta-analysis, involving 2,557 healthy subjects (mean age varies from 21 to 86 years). Long-term ET induced significantly decreased in the levels of interleukin-6 (IL-6) (SMD -0.16, 95% CI -0.30 to −0.03, *p* = 0.017), C-reactive protein (CRP) (SMD -0.18, 95% CI -0.31 to −0.06, *p* = 0.005), as well as tumor necrosis factor alpha (TNFα) (SMD -0.43, 95% CI -0.62 to −0.24, *p* < 0.001). Subgroup analysis revealed that Long-term ET conducted for more than 12 weeks and exercise of moderate intensity had greater anti-inflammatory effects. Meta-regression analysis showed that the reduction in CRP level induced by long-term ET was weakened by increasing exercise intensity.

**Conclusion:**

Long-term ET induced significant anti-inflammatory effects in healthy subjects. Long-term ET-induced anti-inflammatory effects were associated with exercise of moderate intensity and training conducted for more than 12 weeks.

**Systematic Review Registration:** https://www.crd.york.ac.uk/PROSPERO/# myprospero, PROSPERO, identifier CRD42022346693.

## Introduction

The global physical inactivity of approximately 27.5% of adults (Guthold et al., 2018) and 81% of teenagers (Guthold et al., 2020) increases 6–10% risk of chronic diseases, premature death (Ozemek et al., 2019) and the risk of dementia (Voss et al., 2019). Lack of physical activity is one of the most common causes of chronic low-grade systemic inflammation, which is related to metabolic syndrome, type 2 diabetes, non-alcoholic fatty liver disease, cardiovascular disease, cancer and other diseases (Furman et al., 2019). Exercise training (ET) is a non-pharmacological tool used to prevent many diseases in which the immune system plays a critical role (Shinkai et al., 1998; Llamas-Velasco et al., 2016; Nieman and Wentz, 2019). Therefore, special attention should be given to investigate the effect of ET on inflammation, which is one of the most important pathological factors for the occurrence and development of various chronic diseases (Rapa et al., 2019; Steven et al., 2019).

Low-grade chronic inflammation is a key physiopathological component of many chronic diseases including cancer (Ritter and Greten, 2019), obesity (Iyengar et al., 2016), type 2 diabetes mellitus (T2DM) (Pradhan et al., 2001), atherosclerosis (Hansson, 2005), and Alzheimer’s diseases (Heneka et al., 2015). Theoretically, ET plays an anti-inflammatory role by reducing visceral fat, promoting anti-inflammatory cytokines production from contracting muscle (Pedersen et al., 2001; Petersen and Pedersen, 2005; Pedersen and Febbraio, 2008), and inducing inflammatory cascades by inhibiting the expression of Toll like receptors on monocytes and macrophages (Flynn and McFarlin, 2006). In practice, there is a dose–response relationship between ET and immune response (Garber et al., 2011). For example, exercise of high intensity has a pro-inflammatory effect (de Lucas et al., 2014; Lindsay et al., 2015), while exercise of moderate intensity plays an anti-inflammatory role (Paolucci et al., 2018). Although observational studies (Wannamethee et al., 2002; Majka et al., 2009) strongly support the anti-inflammatory effect of ET, there is no consistency in data obtained from randomized controlled trials (RCTs) (Marcell et al., 2005; Irandoust and Taheri, 2018; Irandoust et al., 2022), and results from large-scale RCTs are limited and inconclusive. Therefore, there is need to establish the effect of ET on inflammation as well as the optimum protocols for ET-induced anti-inflammatory effects.

Previous meta-analyzes focused on the effects of ET on inflammatory biomarkers in diseased individuals (Wu et al., 2022; Xing et al., 2022), as well as the acute effects of a single ET on inflammatory biomarkers (Neefkes-Zonneveld et al., 2015), and their research findings might be influenced by specific diseases. As a result, there have been no conclusive reports on the effects of exercise on inflammation in healthy individuals. There is therefore need to conduct comprehensive meta-analysis of existing RCTs to: 1) determine the effect of long-term ET on biomarkers of inflammation [e.g., interleukin 6 (IL-6), C-reactive protein (CRP), and tumor necrosis factor alpha (TNFα)] under normal physiological conditions in healthy subjects, 2) investigate how training protocols and characteristics of subjects influence the outcomes, and 3) suggest scientifically proven and credible exercise regimens for healthy people.

## Materials and methods

### Literature search

This study registry on PROSPERO (ID: CRD 42022346693). This meta-analysis was conducted in compliance with the Preferred Reporting Items for Systematic Reviews and Meta-Analysis (PRISMA) guidelines (Page et al., 2021). Relevant articles published between 1980 and May 1st, 2023 were retrieved from PubMed, Web of Science, Cochrane Library, Embase, PsycINFO, Cumulated Index to Nursing and Allied Health Literature (CINAHL), and SPORTDiscus, based on the Population, Intervention, Comparator, Outcome and Study design (PICOS) framework. The following search strategy was used: (“Exercise training” OR “Physical Exercise” OR “Exercise Therapy”) AND (“Inflammation” OR “Interleukins” OR “Tumor Necrosis Factors” OR “Cytokines”) AND “randomized controlled trial.” Detailed search strategies are shown in Supplementary Table S1.

### Study selection

Two researchers (YHW and JWT) independently screened titles and abstracts, then reviewed full-text for eligibility. The third researcher (MC) arbitrated any discrepancies to reach consensus. We also conducted a manual search for references to included articles and relevant review articles. Research and review articles were included in the study based on the following inclusion criteria: (Guthold et al., 2018) the volunteers were healthy individuals; (Guthold et al., 2020) the intervention of interest was any type of long-term ET of any intensity, frequency; (Ozemek et al., 2019) the comparisons involved ET versus non-exercise control or ET plus other intervention versus other intervention only; (Voss et al., 2019) the outcomes of interest were inflammatory biomarkers in plasma or serum; (Furman et al., 2019) parallel or crossover randomized controlled trials; and (Nieman and Wentz, 2019) articles published after 1980. The exclusion criteria included (Guthold et al., 2018) studies involving sick individuals; (Guthold et al., 2020) The intervention was the acute effect of one-time ET on inflammatory biomarkers; (Ozemek et al., 2019) studies lacking baseline data or data on the final assessment of outcomes in both intervention group and comparators used to calculate mean changes of treatment ± SD.

### Data extraction and quality assessment

Data extraction and quality evaluation were conducted by two trained researchers (YHW and JWT) independently. The data extracted included the first author’s surname, publication year, study design, study location, sample size, participants age and gender, baseline body mass index (BMI) of participants, ET intervention (duration, type, intensity, frequency), and outcomes of reported biomarkers. Research articles that contained two or more ET intervention strata (e.g., different type, intensity, frequency or duration of ET), were analyzed as separate trials. All types of ET (i.e., aerobic exercise, resistance exercise and multicomponent exercise) were included in this review. Intensity of ET was classified by maximal heart rate (HRmax), maximal oxygen uptake (VO_2_peak) and repetition maximum (RM) according to the guidelines by American College of Sports Medicine (Haskell et al., 2007). The methodological quality and risk of bias of each included study were evaluated using the Physiotherapy Evidence Database (PEDro) scale (Maher et al., 2003). External validity (item 1: Eligibility criteria and source), internal validity (items 2 to 9: Random allocation; Concealed allocation; Baseline comparability; Blinding of participants; Blinding of therapists; Blinding of assessors; Adequate follow-up (>85%); Intention-to-treat analysis) and statistical reporting (items 10 and 11: Between-group statistical comparisons; Reporting of point measures and measures of variability) are covered by the PEDro scale’s eleven items. Items were rated yes or no (1 or 0) based on whether the study obviously meets the criterion. The cumulative PEDro score ranged from 0 to 10 and is calculated by adding the ratings of items 2 to 11. The higher the score, the superior the methodological quality, and the PEDro scale’s scores of 4 were considered “poor,” 4 to 5 were considered “fair,” 6 to 8 are considered “good,” and 9 to 10 were considered “excellent” (Cashin and McAuley, 2020).

The Grading of Recommendation, Assessment, Development and Evaluation (GRADE) system was used to assess the evidence level of each outcome (Goldet and Howick, 2013). According to the GRADE guidelines, study design dictates baseline quality of the evidence (RCTs were initially defined as high quality) but other factors could decrease (e.g., unexplained heterogeneity) or increase (e.g., a large magnitude of effect) the quality of the studies (Goldet and Howick, 2013). Discrepancies were resolved through discussion with the third reviewer (MC).

### Statistical analysis

There were differences among the included studies in study participants, ET protocols employed and the measurement of biomarkers. As a result, the random effects model was used to pool estimates of net changes (changes of intervention group minus changes of control group) in the concentrations of biomarkers and the results were presented as standardized mean difference (SMD) with 95% confidence intervals (CIs). Advanced data extraction was used for studies that did not directly provide either the baseline or final mean and standard deviation (SD) of outcomes, according to the protocol proposed by Wan et al. (2014).

First, a primary meta-analysis was conducted to establish the overall effect of ET on each biomarker. Then sensitivity analysis was conducted by using a leave-one-out meta-analysis (LOOM) to test the robustness of the primary results and the influence of each report on the effect or heterogeneity. Sources of potential heterogeneity were identified by carrying out subgroup analyzes based on region, gender, age of participants, baseline BMI of participants, duration of intervention, type of ET and intensity of ET (only conducted if more than six trials reported the same outcomes), and subgroup stratification was based on the characteristics of the included studies and also referred to the previous meta-analysis stratification method (Schwingshackl et al., 2014; de Souto Barreto et al., 2019). Difference between groups and sources of heterogeneity were tested using meta-regression analysis. Further meta-regression analyzes were conducted to investigate potential moderators and examine the association between moderators and outcomes, with value of *p* <0.1 being considered as statistically significant. The heterogeneity among studies was tested using Cochrane’s Q test and quantified using I^2^-statistic (Higgins et al., 2003). Presence of heterogeneity was indicated by I^2^ > 50% and *p* value <0.1 for Q test. Begg’s and Egger’s regression tests as well as funnel plots were utilized to assess publication bias, with a value of *p* <0.05 suggesting the presence of bias (Egger et al., 1997). If publication bias was encountered, the trim and fill method was used to remove extremely small studies and recalculate the pooled effect iteratively until the funnel plot was symmetrical (Duval and Tweedie, 2000). All analyzes were performed using STATA version 16.0 (Stata Corp, College Station, TX, United States), with double data input to avoid input errors. *p* < 0.05 was deemed as statistically significant unless specified elsewhere.

## Results

### Flow of study selection

**Figure 1:**
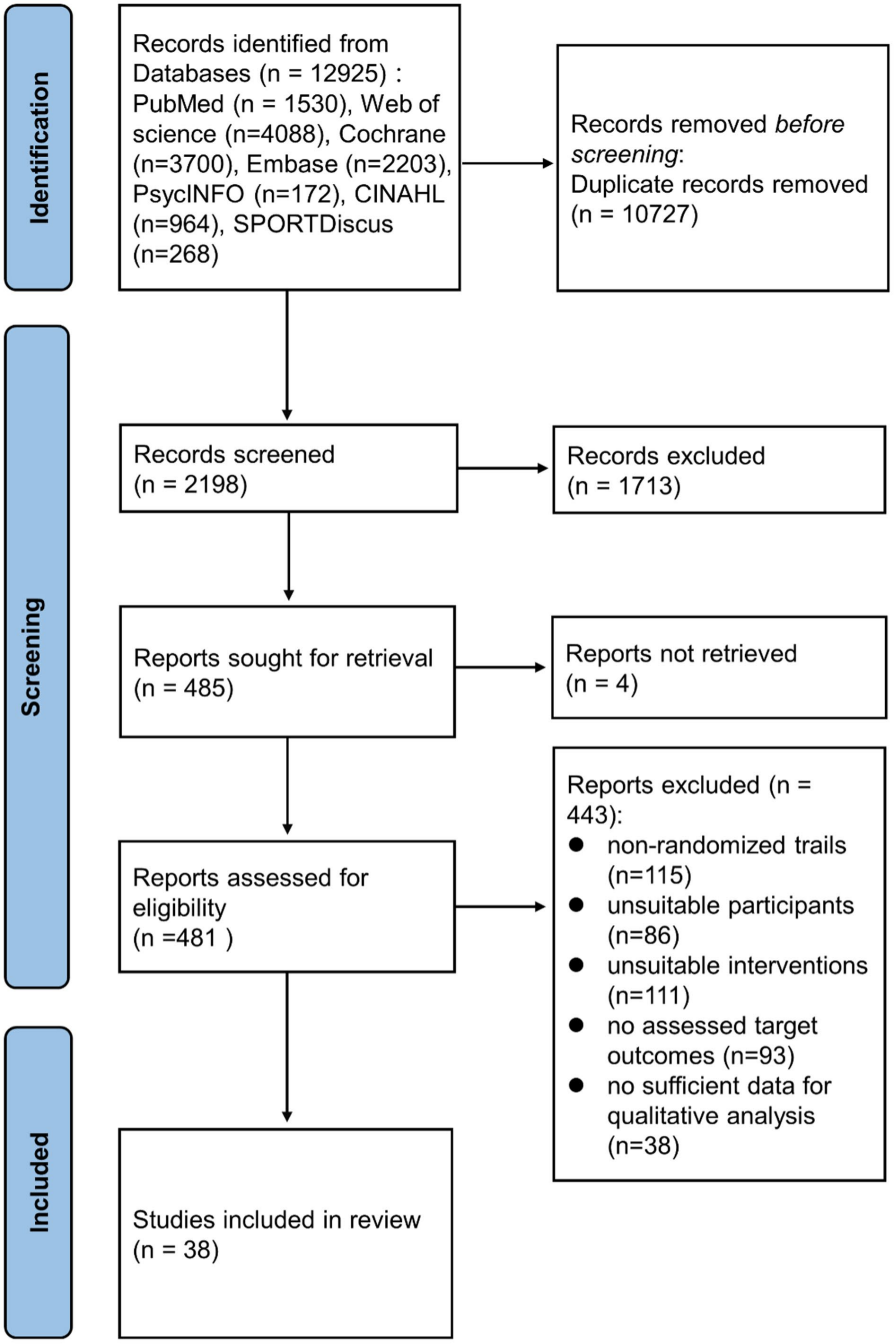
A flowchart showing the procedure used for study selection.

The flowchart depicting the study selection procedure is shown in Figure 1. Initially, 12,925 publications were retrieved, but after excluding the duplicates and screening the titles and abstracts, only 481 articles were left for full-text review. A further 443 articles were eliminated for the following reasons: 115 articles did not involve RCT design, participants of 86 studies were not healthy, 111 articles had improper intervention or control, assessable target outcomes were not reported in 93 articles, and 38 articles lacked sufficient data for quantitative analysis. Finally, 38 studies (Campbell et al., 2008; Nicklas et al., 2008; Levinger et al., 2009; Donges et al., 2010; Martins et al., 2010; Phillips et al., 2010; Lee et al., 2012; Libardi et al., 2012; So et al., 2013; Beltran Valls et al., 2014; Forti et al., 2014; Olesen et al., 2014; Rodriguez-Miguelez et al., 2014; Azizbeigi et al., 2015; Nishida et al., 2015; Strandberg et al., 2015; Alghadir et al., 2016; Buyukyazi et al., 2017; Lira et al., 2017; Ihalainen et al., 2018; Paolucci et al., 2018; Sloan et al., 2018; Tomeleri et al., 2018; Cunha et al., 2019; Ihalainen et al., 2019; Meyer et al., 2019; Urzi et al., 2019; Bagheri et al., 2020; Masala et al., 2020; Ward et al., 2020; Biesek et al., 2021; Dani et al., 2021; Kirk et al., 2021; Nikniaz et al., 2021; Omar et al., 2021; Qi et al., 2021; Tang et al., 2021; Griffen et al., 2022) involving 2,557 participants were included in the final meta-analysis.

### Qualities of included studies and outcome measure evidences by GRADE system

The quality of the methodology of three studies (Olesen et al., 2014; Dani et al., 2021; Griffen et al., 2022) was rated as excellent according to the PEDro scale (scores ≥9), while the remaining 35 studies were rated as good quality (6–8 scores) mainly due to lack of blinding (Table 1). According to the GRADE system, the quality of evidence for IL-6, CRP were moderate, and TNFα was low (Supplementary Table S2).

**Table 1:** Characteristics of included studies in this meta-analysis (38 studies).

Study	Design	Country	Subject	Comparators	Interventions	Duration of intervention	Biomarkers Outcomes	Quality^a^
Alghadir et al. (2016)	RP	Saudi Arabia	Healthy subjects *(N = 100)*	*Control (N = 50)*: Not described	*Aerobic Exercise (N = 50)*: stretching exercises and walking (5–10 min); treadmill, bicycle, and stair training (45–60 min). *Intensity:* 30–45% of VO_2_max *Frequency:* 3 times / wk	24 wk	CRP	Good
Azizbeigi et al. (2015)	RP	Iran	Male physical education students *(N = 30)*	*Control (N = 10)*: Not described	*Resistance Exercise (N = 20)*: upper body and lower body exercises: chest press, lat pull down, leg extension and flexion, biceps and triceps curls, squats, and sit-ups. *Intensity:* 65–70% (moderate intensity group, N = 10) / 85–90% (high intensity group, N = 10) of 1RM. *Frequency:* 3 times / wk.	8 wk	IL-6, TNFα	Good
Bagheri et al. (2020)	RP	Iran	overweight middle-aged men *(N = 30)*	*Control (N = 15)*: Not described	*Aerobic Exercise (N = 15)*: circuit training, fast walking or jogging. *Intensity:* moderate intensity (40–59% of the heart rate reserve). *Frequency:* 3 times / wk	8 wk	IL-6, CRP, TNFα	Good
Biesek et al. (2021)	RP	Brazil	pre-frail older women *(N = 72)*	*Control (N = 36)*: Not described	*Multicomponent Exercise (N = 36)*: The training was divided into a warm-up (10 min), neuromotor exercises (10 min), resistance exercises (20 min), and a cool-down (10 min). exergames training (N = 18), exergames combined with protein supplementation (N = 18). *Intensity:* moderate intensity *Frequency:* 2 times / wk.	12 wk	IL-6	Good
Buyukyazi et al. (2017)	RP	Turkey	Pre-menopausal women *(N = 30)*	*Control (N = 8)*: Non exercising	*Aerobic Exercise (N = 22)*: warm-up (5 min); walking (30–50 min); cool-down (5 min). *Intensity:* 50–55% (moderate tempo walking group) / 70–75% (brisk walking group) of HR reserve *Frequency:* 3 days / wk	8 wk	IL-6	Good
Campbell et al. (2008)	RP	United States	Sedentary men and women *(N = 188)*	*Control (N = 97)*: Not to change their exercise habits	*Aerobic Exercise (N = 91)*: 60 min / d *Intensity:* 60–85% of HRmax *Frequency:* 6 days / wk	48 wk	CRP	Good
Cunha et al. (2019)	RP	Brazil	untrained older women *(N = 48)*	*Control (N = 23)*: Maintain daily activities	*Resistance Exercise (N = 25)*: The exercises were performed in the following order: chest press, horizontal leg press, seated row, knee extension, preacher curl (free weights), leg curl, triceps pushdown, and seated calf raise. *Intensity:* / *Frequency:* 3 times / wk	12 wk	CRP	Good
Dani et al. (2021)	RP, Db	Brazil	healthy elderly women *(N = 19)*	*Control (N = 9)*: consumed 400 mL of grape juice per day	*Multicomponent Exercise (N = 10)*: resistance and aerobic training (60 min each session). *Intensity:* moderate intensity *Frequency:* 2 times / wk	4 wk	IL-6	Excellent
Donges et al. (2010)	RP	Australia	Sedentary subjects *(N = 102)*	*Control (N = 26)*: Maintained sedentary lifestyle and dietary patterns	*Resistance Exercise (N = 35)*: warm-up (5 min); pulley-weight machine exercises (30–50 min); stretching (5 min). *Intensity:* 70–75% of 10RM *Frequency:* 2–3 sessions / wk. *Aerobic Exercise (N = 41)*: warm-up (5 min); cycling exercise (30–50 min); stretching (5 min). *Intensity:* 70–75% of HRmax *Frequency:* 2–3 sessions / wk	10 wk	IL-6、CRP	Good
Forti et al. (2014)	RP	Belgium	Elderly volunteers *(N = 40)*	*Control (N = 20)*: Maintain daily activity levels	*Resistance Exercise (N = 20)*: warm-up; progressive strength training; muscle stretching (60 min). *Intensity:* 50–80% of 1RM *Frequency:* 3 sessions / wk	12 wk	IL-6	Good
Griffen et al. (2022)	RP, Db	United Kingdom	Healthy older men *(N = 36)*	*Control (N = 18)*: not alter their habitual diet or physical activity levels	*Resistance Exercise (N = 18)*: 5-min warm up, followed by 3 sets of leg press, lateral row, hamstring curl, chest press, leg extension and shoulder press (in that order) on fixed RE machines *Intensity:* 60(first 4 weeks)-80% of 1RM *Frequency:* 2 sessions / wk	12 wk	IL-6, CRP, TNFα	Excellent
Ihalainen et al. (2018)	RP	Finland	Healthy young men *(N = 49)*	*Control (N = 18)*: Keep their dietary intake constant	*Multicomponent Exercise (N = 31)*: cycle ergometer (30–50 min); resistance training (30–50 min). *Intensity:* low to high *Frequency:* 2–3 (aerobic and resistance training consecutively in the same training session group, N = 16) / 4–6 (on alternating days group, N = 15) days / wk	24 wk	IL-6, CRP, TNFα	Good
Ihalainen et al. (2019)	RP	Finland	Healthy older individuals *(N = 92)*	*Control (N = 18)*: Maintain their normal physical activity	*Resistance Exercise (N = 74)*: whole-body strength training *Intensity:* 70–90% 1RM *Frequency:* 1(N = 24) / 2 (N = 24) / 3 (N = 26) times / wk	24 wk	IL-6, CRP	Good
Kirk et al. (2021)	RP	United Kingdom	Community-dwelling older adults *(N = 100)*	*Control (N = 54)*: maintain their physical activity and habitual food intake	*Resistance Exercise (N = 46)*: included leg, chest, calf, and shoulder presses; seated row; back extensions and bicep curl; and two sets to fatigue of each exercise with 3 min break *Intensity:* / *Frequency:* 3 times / wk	16 wk	IL-6, CRP, TNFα	Good
Lee et al. (2012)	RP	Korea	Healthy and untrained females *(N = 22)*	*Control (N = 7)*: No exercise training	*Aerobic Exercise (N = 15)*: treadmill running (volume equated relative to kilograms of body weight). *Intensity:* 50% (low-intensity group, N = 8) / 70% (high-intensity group, N = 7) of VO_2_max *Frequency:* 3–5 sessions / wk	14 wk	IL-6, CRP, TNFα	Good
Levinger et al. (2009)	RP	Australia	Individuals with a low number of metabolic risk factors *(N = 25)*	*Control (N = 13)*: Non exercise	*Resistance Exercise (N = 12)*: resistance training including chest press, leg press, lateral pull-down, triceps push-down, knee extension, seated row and biceps curl. *Intensity:* gradually increased from 40–50% of 1RM to 75–85% of 1RM *Frequency:* 3 days / wk	10 wk	IL-6, CRP, TNFα	Good
Libardi et al. (2012)	RP	Brazil	Inactive male subjects *(N = 47)*	*Control (N = 13)*: Maintain their previous nutritional patterns	*Resistance Exercise (N = 11)*: upper and lower body exercises (60 min) *Intensity:* 8–10 repetition maximum *Aerobic Exercise (N = 12)*: walking or running (60 min) *Intensity:* 55–85% of VO_2_peak *Multicomponent Exercise (N = 11)*: performed resistance exercise (30 min) and aerobic exercise (30 min) in the same session. *Frequency:* 3 sessions / wk	16 wk	IL-6, CRP, TNFα	Good
Lira et al. (2017)	RP	Brazil	Recreationally active men *(N = 30)*	*Control (N = 10)*: Performed no intervention	*Aerobic Exercise (N = 20)*: warm-up (5 min); 5 km run intermittently: run (1 min) + recovery (1 min) (high-intensity intermittent training group) / 5 km run continuously (steady state training group) *Intensity:* 100% (high-intensity intermittent training group, N = 10) / 70% (steady state training group, *N* = 10) of maximal aerobic speed *Frequency:* 3 times / wk	5 wk	IL-6, TNFα	Good
Martins et al. (2010)	RP	Portugal	Women and men aged >64 years *(N = 45)*	*Control (N = 13)*: Without formal exercise but maintaining their lifestyle routines	*Aerobic Exercise (N = 18)*: warm-up; aerobic exercise; cool-down (45 min). *Intensity:* from 40–50% increasing to 71–85% of HR reserve *Resistance Exercise (N = 14)*: warm-up; strength training based on callisthenic exercises and elastics bands; cool-down (45 min). *Intensity:* moderate *Frequency:* 3 times / wk	16 wk	CRP	Good
Masala et al. (2020)	RP	Italy	Post-menopausal women *(N = 115)*	*Control (N = 58)*: Participants received general advice on healthy dietary and physical activity patterns	*Aerobic Exercise (N = 57)*: working, biking, etc. (<1 h / day). *Intensity:* moderate *Frequency:* everyday *Resistance Exercise (N = 57)*: strenuous activity *Frequency:* 1 h / wk	96 wk	IL-6, CRP, TNFα	Good
Meyer et al. (2019)	RP	United States	Healthy middle-aged and older adults *(N = 258)*	*Control (N = 132)*: observational control	*Aerobic Exercise (N = 126)*: cognitive behavioral concerns (1.5 h) + warm-up; aerobic exercise; cool-down (1 h) + half-day retreat. *Intensity:* moderate *Frequency:* 1 session / wk	8 wk.,25 wk	IL-6, CRP	Good
Nicklas et al. (2008)	RP, Sb	United States	Non-disabled, community-dwelling men and women *(N = 369)*	*Successful Aging (N = 186)*: health education; stretching (5-10 min). *Frequency:* 1 session / wk. (1–26 wk); 1 session/month (27–48 wk).	*Multicomponent Exercise (N = 183)*: warm-up; combination of aerobic, strength, balance, and flexibility exercises; cool-down. *Intensity:* moderate *Frequency:* 3–5 sessions / wk	48 wk	IL-6, CRP	Good
Nikniaz et al. (2021)	RP	Iran	healthy male smokers *(N = 40)*	*Control (N = 40)*: follow their daily CS behavior and regular diet	*Aerobic Exercise (N = 40)*: performed aerobic running (30 min). *Intensity:* 50–60%(first two weeks) to 60–70% HR max. *Frequency:* 3 times / wk	4 wk	IL-6, TNFα	Good
Nishida et al. (2015)	RP	Japan	Healthy post-menopausal females *(N = 62)*	*Control (N = 31)*: Maintain their normal lifestyle	*Aerobic Exercise (N = 31)*: bench step exercise (10–20 min). *Intensity:* corresponding to lactate threshold *Frequency:* 3 times / day	12 wk	IL-6, TNFα	Good
Olesen et al. (2014)	RC, Db	Denmark	Physically inactive healthy male subjects *(N = 20)*	*Placebo (N = 7)*: Continue their habitual lifestyle	*Multicomponent Exercise (N = 13)*: high-intensity interval spinning training (cycle ergometer); full-body circuit training. *Intensity:* high *Frequency:* 2 times / wk.; 1 time / wk.	8 wk	IL-6, CRP, TNFα	Excellent
Omar et al. (2021)	RC	Malaysia	Healthy young man *(N = 70)*	*Control (N = 34)*: no change in walking	*Aerobic Exercise (N = 36)*: minimum target: 8,000 steps/day *Intensity:* low *Frequency:* everyday	12 wk	IL-6, CRP, TNFα	Good
Paolucci et al. (2018)	RP	Canada	Healthy young adults *(N = 55)*	*Control (N = 18)*: Remain sedentary	*Aerobic Exercise (N = 37)*: High intensity interval training group (*N* = 19): warm-up (3 min); cycling at ten 60-s high-intensity intervals with ten 60-s active recovery intervals (20 min); cool-down (2 min). *Intensity:* 80% maximum wattage Moderate continuous training group (*N* = 18): warm-up (3 min); cycling at steady state (27.5 min); cool-down (2 min). *Intensity:* 40% maximum wattage *Frequency:* 3 times / wk	6 wk	IL-6, CRP, TNFα	Good
Phillips et al. (2010)	RP	United States	Healthy sedentary post-menopausal women *(N = 35)*	*Control (N = 7)*: Rested quietly during experimental period	*Resistance Exercise (N = 28)*: 3 sets of 8 repetitions of resistance training; 2-min rest between sets. *Intensity:* 70–80% of 1RM *Frequency:* 3 times / wk.	10 wk	IL-6, TNFα	Good
Qi et al. (2021)	RP	China	Cognitively healthy older people *(N = 48)*	*Control (N = 26)*: received group-based stretching	*Aerobic Exercise (N = 22)*: consisting of warm-up movement and nine movements involving the stretching of arms and legs, the turning of the torso, and relaxing *Intensity:* low *Frequency:* 2 times / wk.	12 wk	IL-6	Good
Rodriguez-Miguelez et al. (2014)	RP	Finland	Healthy participants *(N = 26)*	*Control (N = 10)*: Followed their daily routines	*Resistance Exercise (N = 16)*: warm-up (10 min); three resistance exercise (leg press, biceps curl, pec deck). *Intensity:* 60–80% of 1RM *Frequency:* 2 sessions / wk	10 wk	IL-6, CRP	Good
Sloan et al. (2018)	RP	United States	Healthy young adults *(N = 103)*	*Control (N = 58)*: Wear the step counter (so do exercise group); maintain sedentary lifestyle	*Aerobic Exercise (N = 45)*: warm-up + cool-down (10–15 min); workout (30–40 min). *Intensity:* 55–75% of HRmax *Frequency:* 4 sessions / wk	12 wk	IL-6, TNFα	Good
So et al. (2013)	RP	Korea	Healthy elderly *(N = 40)*	*Control (N = 22)*: Continue their habitual lifestyle	*Resistance Exercise (N = 18)*: One bout of training (60 min) was composed of three steps: warm-up for 15 min, exercise for 40 min, and cool-down for 5 min. *Intensity:* low level *Frequency:* 3 times / wk	12wk	IL-6, CRP, TNFα	Good
Strandberg et al. (2015)	RP	Sweden	Healthy older women *(N = 42)*	*Control (N = 21)*: Continue their habitual lifestyle	*Resistance Exercise (N = 21)*: The following exercises were performed: squat, leg extension, leg press, seated row, and pull down. 5 min of core stability exercises and seven squat jumps were included. *Intensity:* workload of 75–85% 1 RM (8–12 reps/set) *Frequency:* 2 times / wk	24 wk	CRP, TNFα	*Good*
Tang et al. (2021)	RP	China	Young adults *(N = 26)*	*Control (N = 14)*: calorie restriction	*Multicomponent Exercise (N = 12)*: 90 min a time (20 min RS plus 10 min rest, with 3 repetitions) *Intensity:* high *Frequency:* 3 times / wk	8 wk	IL-6, CRP, TNFα	Good
Tomeleri et al. (2018)	RP	Brazil	Older women *(N = 45)*	*Control (N = 23)*: Did not perform any type of physical exercise	*Resistance Exercise (N = 22)*: 3 sets of 10–15 repetitions; rest between sets (1–2 min) / exercise (2–3 min) *Intensity:* not mentioned *Frequency:* 3 times / wk	18 wk	IL-6, CRP, TNFα	Good
Urzi et al. (2019)	RP	Slovenia	Female nursing home *(N = 20)*	*Control (N = 9): did not receive any placebo or treatment*	*Resistance Exercise (N = 11)*: warmup of 10 min and 35 to 40 min of 8 resistance exercisesd chair squats; band seated: biceps curl, seated row, knee extension, leg press and hip abduction; and standing behind the chair: knee flexion and calf rise. *Intensity:* moderate level *Frequency:* 3 times / wk	12 weeks	CRP	Good
Beltran Valls et al. (2014)	RP	Italy	Old community-dwelling people *(N = 23)*	*Control (N = 10)*: Maintained the usual lifestyle habits	*Resistance Exercise (N = 13)*: warm-up (10 min); 3–4 sets of 10–12 repetitions; rest between sets (2 min) / exercise (3 min). *Intensity:* 70% of 1RM *Frequency:* not mentioned	12 wk	IL-6, TNFα	Good
Ward et al. (2020)	RP	Sweden	Postmenopausal women *(N = 55)*	*Control (N = 29)*	*Resistance Exercise (N = 26)*: chest press, leg press, seated row, leg curl, latissimus dorsi pull-down, leg extension, crunches and back raises. *Intensity:* 8 repetition-maximum (8 RM) *Frequency:* 3 times / wk	15 wk	CRP, TNFα	Good

a Better methodological quality is indicated by a higher PEDro score (9 to 10: excellent; 6 to 8: good; 4 to 5: fair; <4: poor).

CRP, C-reactive protein; Db, double blind; kcal, kilocalories; km, kilometer; HRmax, maximal heart rate; IL-6, interleukin 6; RC, randomized crossover; RM, repetition maximum; RP, randomized-parallel; s, second; SB, single-blinded; TNFα, tumor necrosis factor alpha; VO2max, maximal oxygen uptake; wk, week.

### Characteristics of included studies

Characteristics of studies included in this meta-analysis are shown in Table 1. The final sample consisted of 2,557 participants, with the mean age ranging from 21 to 86 years. Sample sizes ranged from 19 to 369 participants, with a median size of 46. The average baseline BMI value of the participants ranged from 21.7 to 29.9. Different types of ET (aerobic exercise: 15 interventions; resistance exercise: 16 interventions; multicomponent exercise: 7 interventions) were evaluated in the studies.

### Effect of long-term ET on IL-6 levels

**Figure 2:**
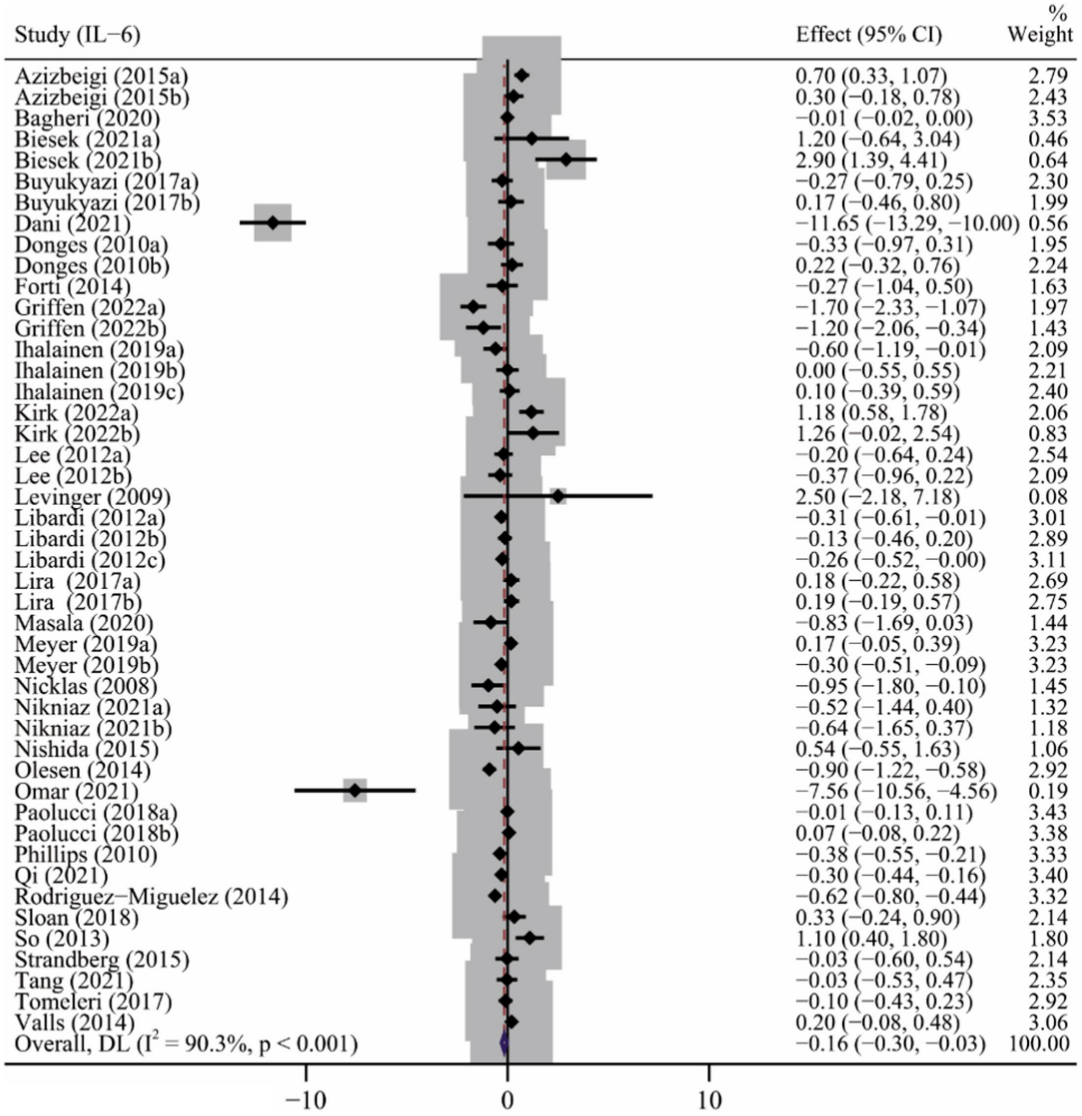
The forest plot of the effect of long-term ET on IL-6 levels.

As shown in Table 2, assessments for IL-6 levels were reported in 32 articles (Nicklas et al., 2008; Levinger et al., 2009; Donges et al., 2010; Phillips et al., 2010; Lee et al., 2012; Libardi et al., 2012; So et al., 2013; Beltran Valls et al., 2014; Forti et al., 2014; Olesen et al., 2014; Rodriguez-Miguelez et al., 2014; Azizbeigi et al., 2015; Nishida et al., 2015; Strandberg et al., 2015; Buyukyazi et al., 2017; Lira et al., 2017; Ihalainen et al., 2018; Paolucci et al., 2018; Sloan et al., 2018; Tomeleri et al., 2018; Ihalainen et al., 2019; Meyer et al., 2019; Bagheri et al., 2020; Masala et al., 2020; Biesek et al., 2021; Dani et al., 2021; Kirk et al., 2021; Nikniaz et al., 2021; Omar et al., 2021; Qi et al., 2021; Tang et al., 2021; Griffen et al., 2022) involving 48 interventions. Results of the meta-analysis showed that long-term ET caused a significant decrease in IL-6 levels (SMD −0.16, 95% CI −0.30 to −0.03), although high heterogeneity was observed among the studies (*p* < 0.001, I^2^ = 90.3%) (Table 2 and Figure 2). Subgroup analysis showed that studies of baseline BMI > 25, female subjects, duration of exercise less than 12 weeks, frequency of less than three times a week, and type of multicomponent exercise were associated with PE-induced reduction in IL-6 levels (Table 2). The result of the Begg’s test (*p* = 0.917), Egger’s test (*p* = 0.091) and funnel plot (Supplementary Figure S1) indicated no significant publication bias in the primary analysis for IL-6 (Table 2).

**Table 2:** Results of sensitivity analysis, subgroup analysis and publication bias stratified by study characteristics.

Outcomes	Interventions	SMD (95% CI)	*P* ^1^	Heterogeneity		*P* ^3^	*P* ^4^	
				*I*^2^ (%)	*P* ^2^		Begg’s value	Egger’s value
**IL-6**								
*Overall*	48	−0.16 (−0.30, −0.03)	**0.017**	90.3	**<0.001**		0.917	0.091
*Gender*						0.303		
Male only	15	−0.19 (−0.44, 0.06)	0.132	86.5	**<0.001**			
Female only	14	**−0.61 (−1.08, −0.13)**	**0.013**	94.3	**<0.001**			
*Age*						0.818		
< 45 years	19	−0.04 (−0.19, 0.12)	0.653	79.5	**<0.001**			
45 ~ 60 years	7	−0.19 (−0.39, 0.02)	0.076	62.1	**0.015**			
**≥** 60 years	20	−0.29 (−0.64, 0.07)	0.112	93.7	**<0.001**			
*Baseline BMI*						0.431		
< 25	13	0.11 (−0.04, 0.26)	0.144	49.9	**0.021**			
>25	33	**−0.30 (−0.50, −0.09)**	**0.005**	92.8	**<0.001**			
*Duration*						0.599		
1 ~ 12 weeks	32	**−0.19 (−0.36, −0.02)**	**0.030**	92.7	**<0.001**			
> 12 weeks	16	−0.13 (−0.34, 0.08)	0.240	63.1	**0.001**			
*Frequency*						0.412		
<3 times/week	13	**−0.84 (−1.37, −0.30)**	**0.002**	90.0	**<0.001**			
**≥**3 times/week	34	−0.03 (−0.22, 0.17)	0.793	66.8	**<0.001**			
*Type of exercise*						0.474		
Aerobic exercise	20	−0.06 (−0.17, 0.05)	0.249	71.9	**<0.001**			
Resistance exercise	18	−0.05 (−0.34, 0.23)	0.712	87	**<0.001**			
Multicomponent exercise	9	−1.28 (−2.56, 0.00)	0.050	97.2	**<0.001**		–	–
*Intensity of exercise*						0.429		
Low	3	−0.93 (−1.95, 0.09)	0.074	91.2	**<0.001**		–	–
Moderate	29	−0.17 (−0.34, 0.01)	0.136	84.1	**<0.001**			
High	12	−0.25 (−0.57, 0.08)	0.769	91.6	**<0.001**			

	Interventions	Net change (95% CI)	*P* ^1^	*I*^2^ (%)	*P* ^2^	*P* ^3^	*P* ^4^	
							Begg’s value	Egger’s value
**CRP**								
*Overall*	39	**−0.18 (−0.31, −0.06)**	**0.005**	88.1	**<0.001**		0.204	0.112
*Gender*						0.852		
Male only	10	−0.17 (−0.43, 0.09)	0.198	86.9	**<0.001**			
Female only	10	−0.12 (0.32, 0.08)	0.244	87.8	**<0.001**		–	–
*Age*						0.242		
< 45 years	10	−0.12 (−0.30, 0.05)	0.177	92.8	**<0.001**			
45 ~ 60 years	10	0.09 (−0.05, 0.23)	0.225	17.9	0.278			
**≥** 60 years	17	**−0.44 (−0.80, −0.09)**	**0.014**	89.7	**<0.001**			
*Baseline BMI*						0.474		
< 25	6	−0.37 (−0.74, 0.01)	0.054	95.5	**<0.001**		–	–
>25	32	**−0.17 (−0.31, −0.03)**	**0.017**	83.8	**<0.001**			
*Duration*						0.435		
1 ~ 12 weeks	17	−0.07 (−0.24, 0.11)	0.439	82.6	**<0.001**			
> 12 weeks	22	**−0.29 (−0.49, −0.10)**	**0.004**	90.4	**<0.001**			
*Frequency*						0.137		
≤3 times/week	8	0.01 (−0.37, 0.38)	0.977	76.1	**<0.001**			
**>**3 times/week	31	**−0.31 (−0.57, −0.05)**	**0.018**	83.7	**<0.001**			
*Type of exercise*						0.699		
Aerobic exercise	15	**−0.23 (−0.41, −0.04)**	**0.017**	89.3	**<0.001**			
Resistance exercise	17	0.02 (−0.25, 0.29)	0.890	78.6	**<0.001**			
Multicomponent exercise	6	−0.27 (−0.60, 0.05)	0.101	93.2	**<0.001**		–	–
*Intensity of exercise*						**0.005**		
Low	3	−1.64 (−3.89, 0.62)	0.155	98.4	**<0.001**		–	–
Moderate	22	**−0.16 (−0.32, −0.01)**	**0.036**	75.2	**<0.001**			
High	8	0.14 (−0.05, 0.23)	0.149	11.4	0.341		–	–
**TNFα**								
*Overall*	32	**−0.43 (−0.62, −0.24)**	**<0.001**	87.8	**<0.001**		0.331	**0.009**
*Gender*						0.102		
Male only	15	**−0.73 (−1.14, −0.31)**	**0.001**	89.9	**<0.001**			
Female only	8	**−0.57 (−0.95, −0.20)**	**0.002**	67.6	**0.003**			
*Age*						0.807		
< 45 years	17	**−0.37 (−0.56, −0.17)**	**<0.001**	82.5	**<0.001**			
45 ~ 60 years	6	**−0.51 (−0.93, −0.10)**	**0.015**	11.3	0.343		–	–
**≥** 60 years	9	−0.35 (−0.90, 0.21)	0.219	93.0	**<0.001**	0.931	–	–
*Baseline BMI*								
< 25	12	**−0.26 (−0.47, −0.06)**	**0.013**	74.4	**<0.001**			
>25	19	**−0.45 (−0.77, −0.13)**	**0.005**	88.1	**<0.001**			
*Duration*						0.600		
1 ~ 12 weeks	20	**−0.48 (−0.72, −0.24)**	**<0.001**	91.1	**<0.001**			
> 12 weeks	12	**−0.34(−0.66, −0.03)**	**0.030**	62.9	**0.002**			
*Frequency*						0.254		
≤3 times/week	3	−1.75 (−4.80, 1.31)	0.262	94.3	**<0.001**			
**>**3 times/week	28	**−0.60 (−0.86, −0.35)**	**<0.001**	73.3	**<0.001**			
*Type of exercise*						0.978		
Aerobic exercise	14	**−0.24 (−0.46, −0.03)**	**0.028**	82.4	**<0.001**			
Resistance exercise	12	**−0.44 (−0.84, −0.04)**	**0.031**	78.6	**<0.001**			
Multicomponent exercise	5	−0.38 (−0.81, 0.05)	0.080	86.4	**<0.001**		–	–
*Intensity of exercise*						0.464		
Low	2	−4.82 (−14.21, 4.56)	0.314	94.9	**<0.001**		–	–
Moderate	18	**−0.57 (−0.89, −0.24)**	**0.001**	81.2	**<0.001**		–	–
High	8	−0.37 (−0.77, 0.04)	0.075	94.2	**<0.001**			

*P*^1^ value for net change; *P*^2^ value for heterogeneity in the subgroup; *P*^3^ value for heterogeneity between groups with meta-regression, analyzed as categorical variables; *P*^4^ value for publication bias (conducted only when trials > 5); significant *p*-values are highlighted in bold prints.

BMI, body mass index; CI, confidence interval; CRP, C-reactive protein; IL-6, interleukin 6; RC, randomized crossover; RP, randomized-parallel; TNFα, tumor necrosis factor alpha.

### Effect of long-term ET on CRP levels

**Figure 3:**
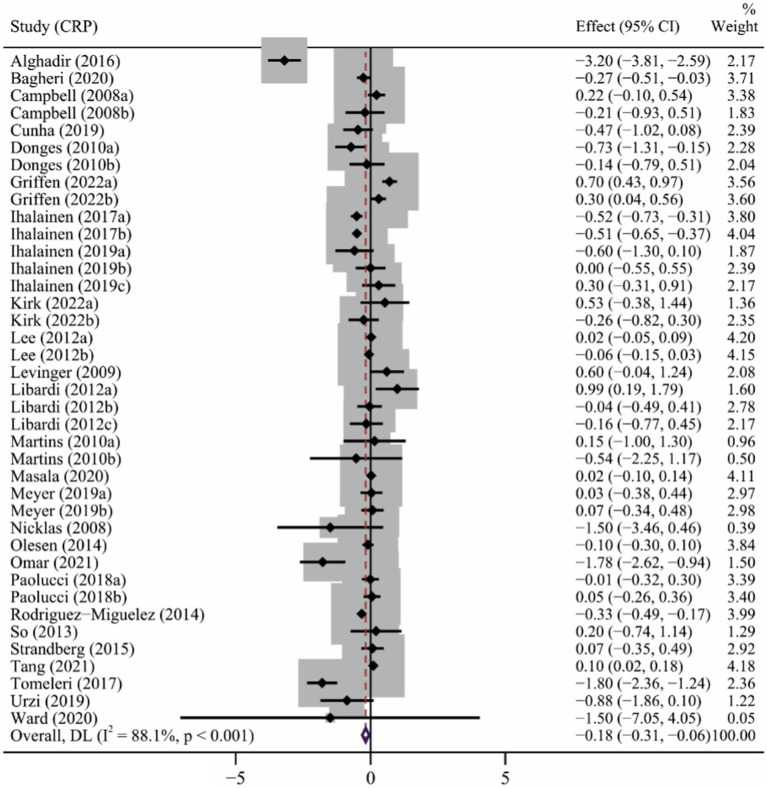
The forest plot of the effect of long-term ET on CRP levels.

A total of 26 articles (Campbell et al., 2008; Nicklas et al., 2008; Levinger et al., 2009; Donges et al., 2010; Martins et al., 2010; Lee et al., 2012; Libardi et al., 2012; So et al., 2013; Olesen et al., 2014; Rodriguez-Miguelez et al., 2014; Strandberg et al., 2015; Alghadir et al., 2016; Ihalainen et al., 2018; Paolucci et al., 2018; Tomeleri et al., 2018; Cunha et al., 2019; Ihalainen et al., 2019; Meyer et al., 2019; Urzi et al., 2019; Bagheri et al., 2020; Masala et al., 2020; Ward et al., 2020; Kirk et al., 2021; Omar et al., 2021; Tang et al., 2021; Griffen et al., 2022) involving 39 interventions explored the effect of long-term ET on CRP levels. Long-term ET induced a significant decrease in CRP levels (SMD -0.18, 95% CI -0.31 to −0.06) compared to placebo/control groups. We found a high heterogeneity across the studies (*p* < 0.001, I^2^ = 88.1%) (Table 2 and Figure 3). Subgroup analyzes showed that studies of participants older than 60 years, baseline BMI > 25, duration of exercise greater than 12 weeks, multicomponent exercise, frequency of three or more times a week, and moderate exercise conferred to a more potent reduction in CRP levels. The result of meta-regression analysis revealed that the intensity of exercise (*p* = 0.005) might be a potential source of heterogeneity (Table 2), which showed that the reduction in CRP level induced by long-term ET was weakened by increasing exercise intensity.

The result of the Begg’s test (*p* = 0.204), Egger’s test (*p* = 0.112) and funnel plot (Supplementary Figure S2) indicated no significant publication bias in the primary analysis for CRP (Table 2).

### Effect of long-term ET on TNFα levels

**Figure 4:**
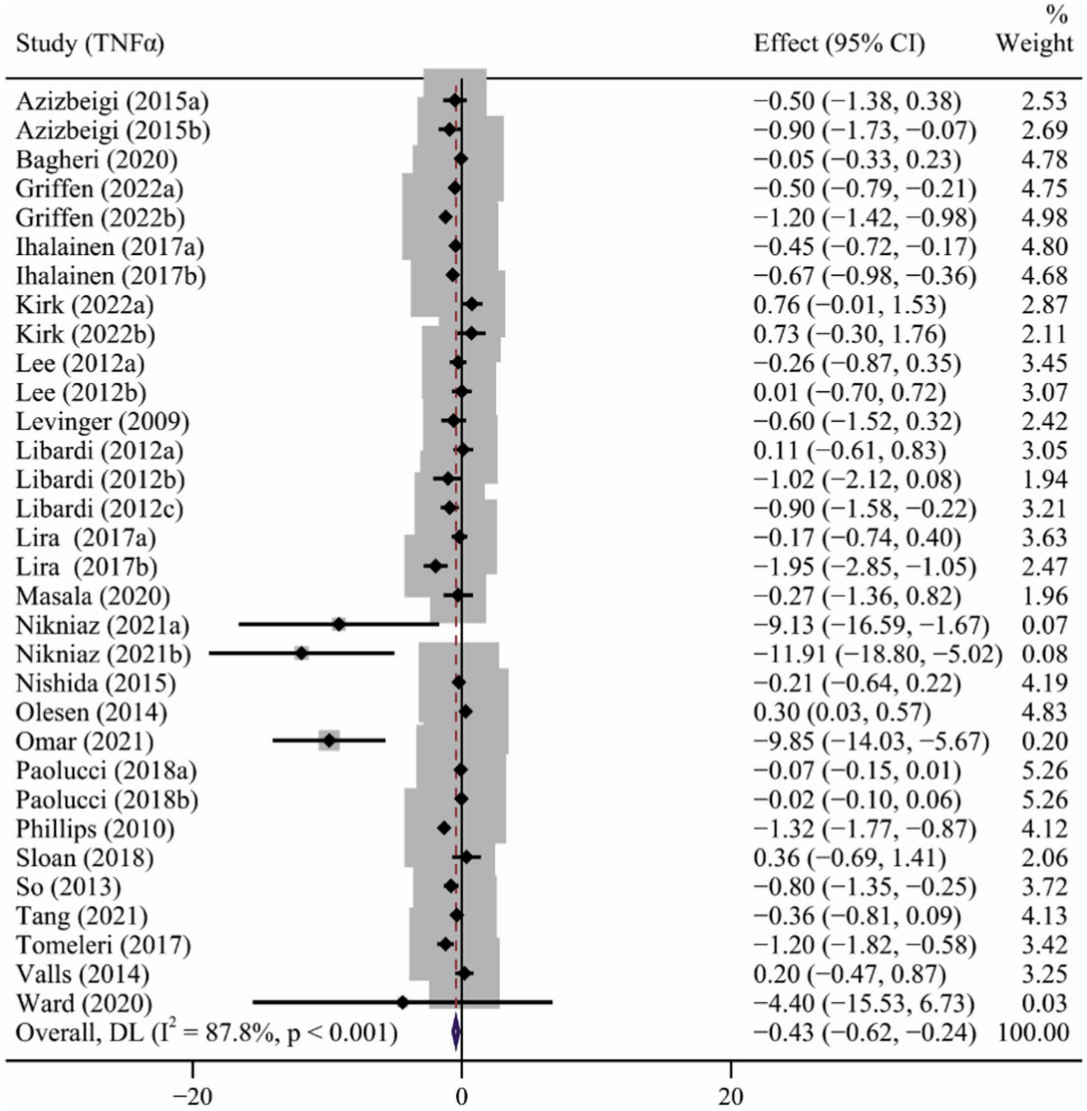
The forest plot of the effect of long-term ET on TNFα levels.

Twenty-two studies (Levinger et al., 2009; Phillips et al., 2010; Lee et al., 2012; Libardi et al., 2012; So et al., 2013; Beltran Valls et al., 2014; Olesen et al., 2014; Azizbeigi et al., 2015; Nishida et al., 2015; Lira et al., 2017; Ihalainen et al., 2018; Paolucci et al., 2018; Sloan et al., 2018; Tomeleri et al., 2018; Bagheri et al., 2020; Masala et al., 2020; Ward et al., 2020; Kirk et al., 2021; Nikniaz et al., 2021; Omar et al., 2021; Tang et al., 2021; Griffen et al., 2022) involving 32 interventions evaluated the effect of long-term ET on TNFα levels. Results of the meta-analysis revealed that long-term ET caused a significant decrease in TNFα levels (SMD −0.43, 95% CI −0.62 to −0.24), with a high heterogeneity across the studies (*p* < 0.001, I^2^ = 87.8%) (Table 2 and Figure 4). Subgroup analyzes demonstrated that subjects with significant reduction in TNFα levels were from studies of participants younger than 60 years, those taking aerobic exercise or resistance exercise, frequency of three or more times a week, and exercise of moderate intensity. Funnel plot (Supplementary Figure S3) and Egger’s test results for TNFα (*p* = 0.009) revealed evidence of publication bias (Table 2).

### Sensitivity analysis

The sensitivity analysis was carried out by leave-one-out meta-analysis (LOOM). The results of the sensitivity analysis indicated that the effect of long-term ET on IL-6 levels was unstable, suggesting that the findings should be handled with caution (Figure 5A). Long-term ET still had a stable significant impact on CRP (Figure 5B) and TNFα (Figure 5C) levels when a single trial was deleted at a time.

**Figure 5:**
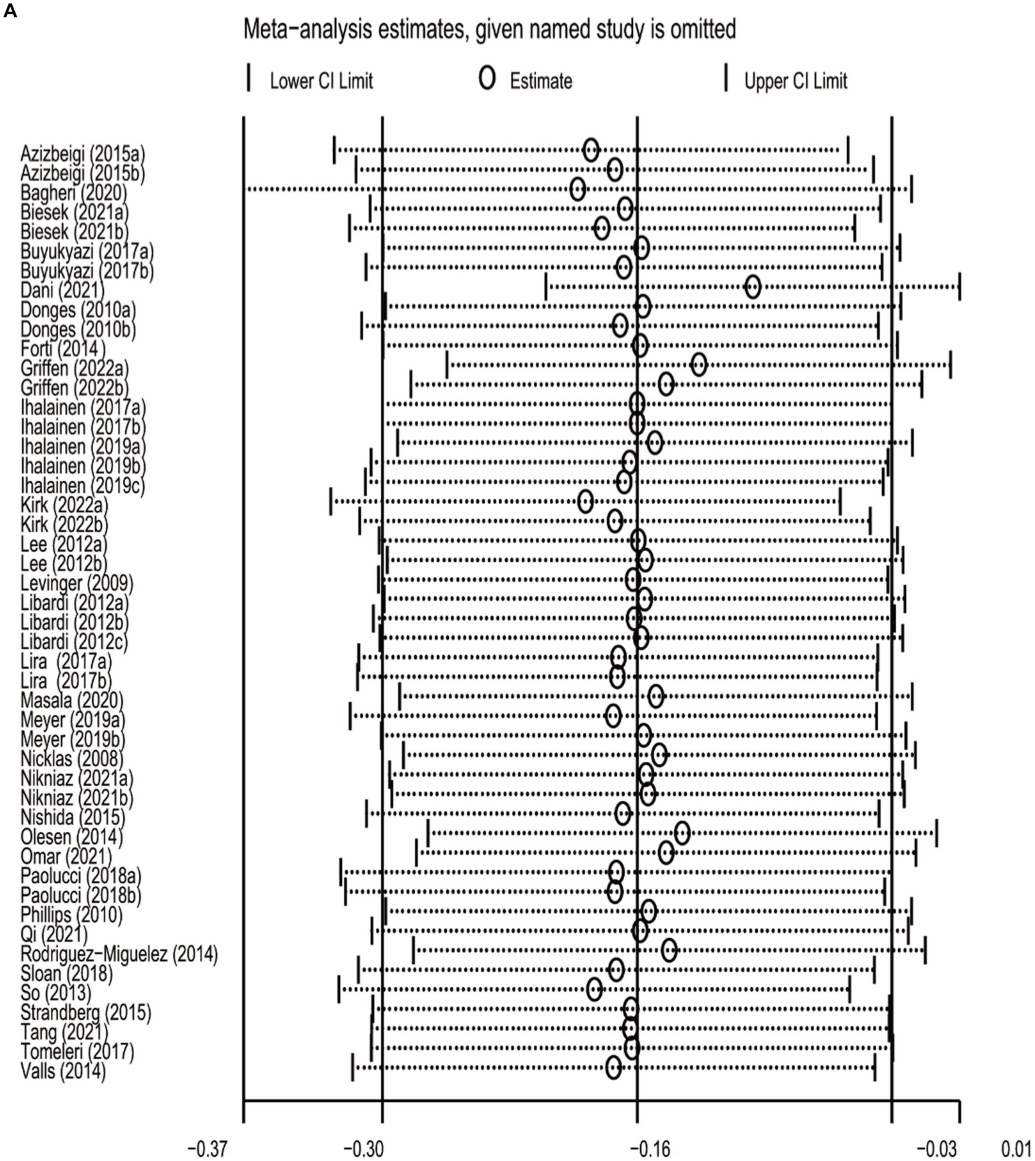


**Figure 5:**
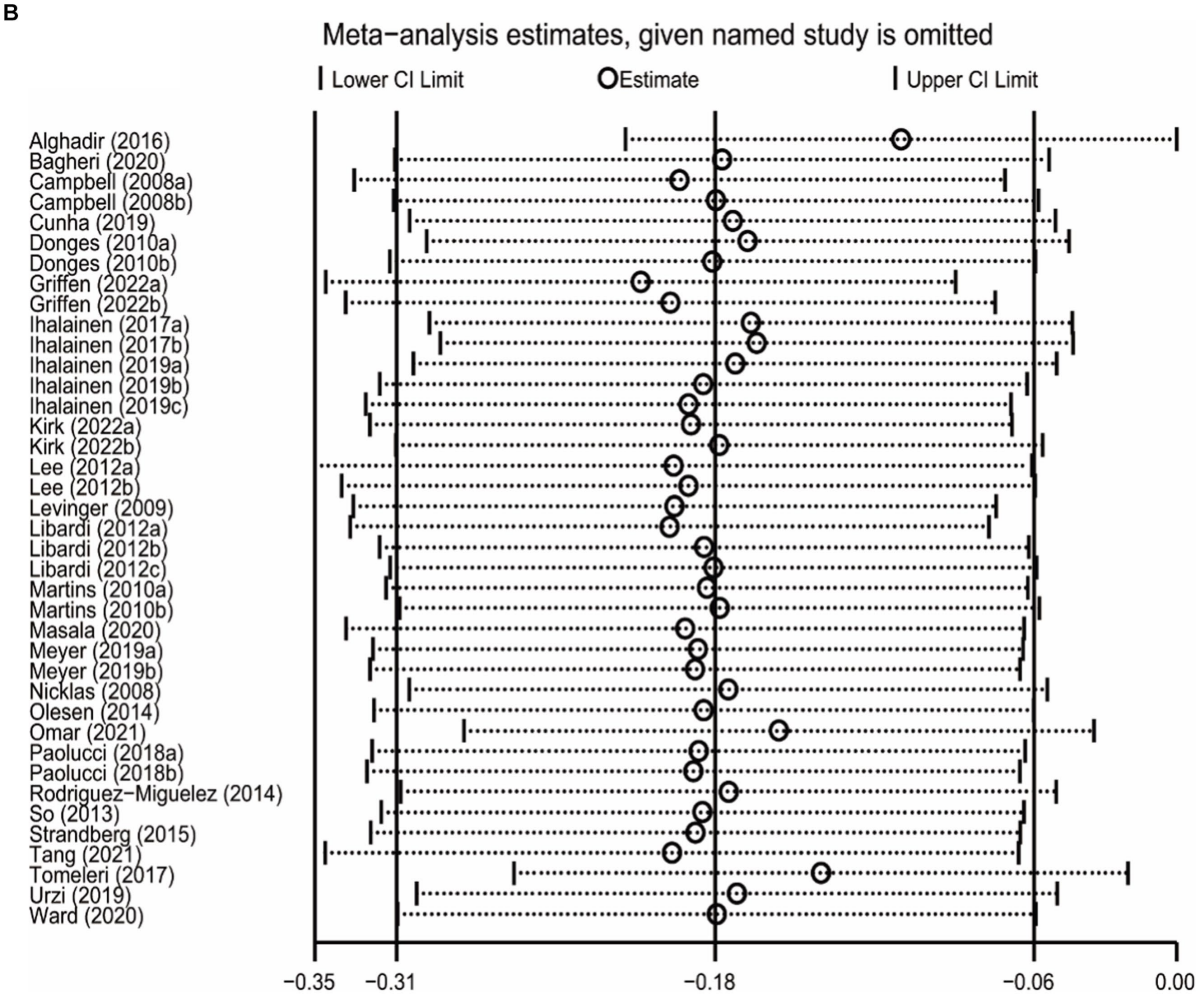


**Figure 5:**
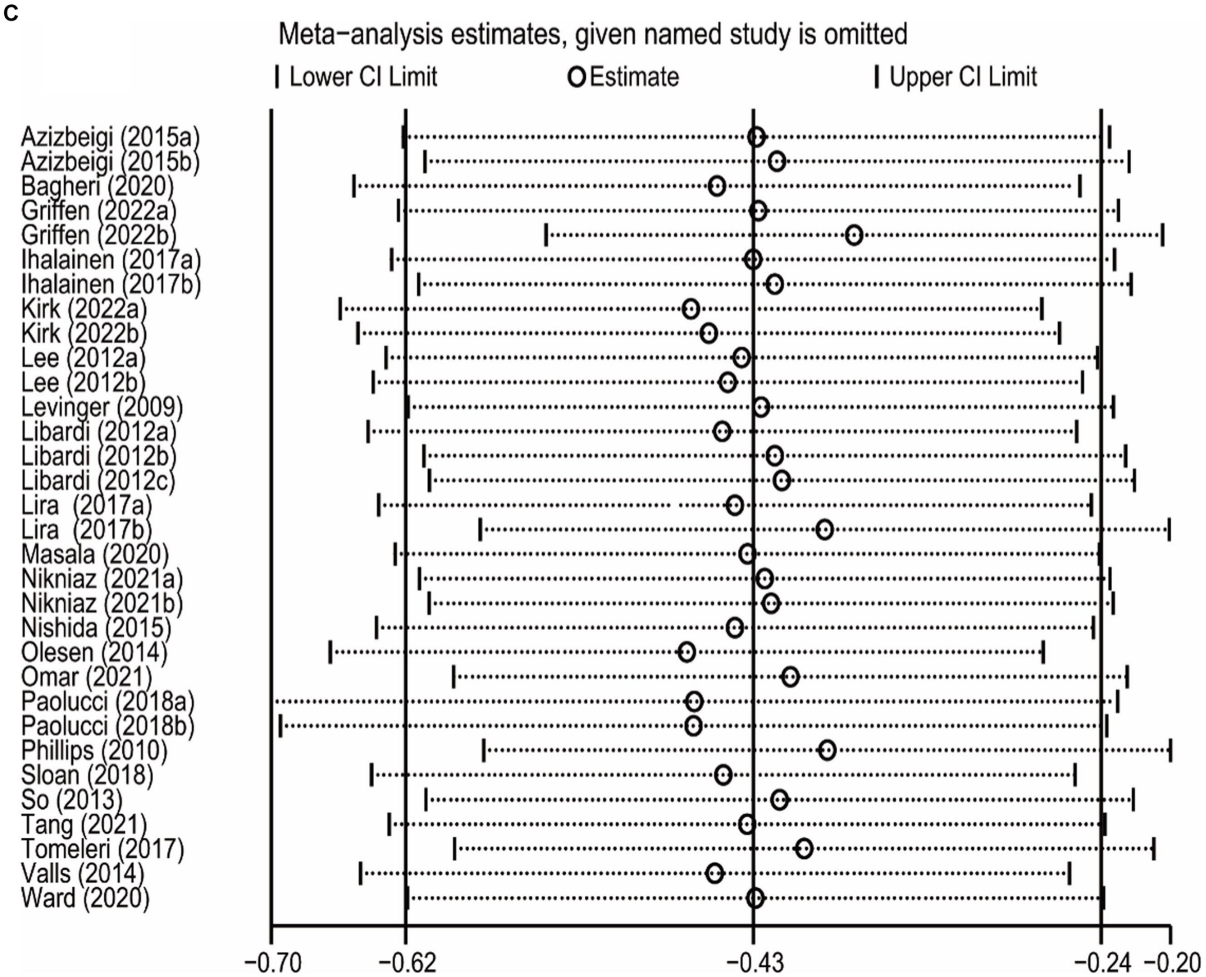
The results of leave-one-out meta-analysis on IL-6 **(A)**, CRP **(B)**, and TNFα **(C)**.

### Adjustment of publication bias

As shown in Table 3, results of pooling estimates indicating publication bias based on Begg’s and Egger’s tests, were recalculated using Duval and Tweedie’s trim and fill method. Long-term ET still induced a significant decrease in TNFα levels (SMD −1.02, 95% CI −1.41 to −0.63).

**Table 3:** Trim and fill adjusted analysis for outcomes with publication bias.

Variables	Interventions (*n*)	Net change (95% CI)		*P*		Adjusted studies
		Before adjusted	After adjusted	Before adjusted	After adjusted	
TNFα	32	−0.43 (−0.62, −0.24)	−1.02 (−1.41, −0.63)	**<0.001**	**<0.001**	5

Significant *p*-values are highlighted in bold prints.

CI, confidence interval; TNFα, tumor necrosis factor alpha.

## Discussion

To the best of our knowledge, this is the first meta-analysis from 38 RCTs to estimate the effect of long-term ET on individual biomarkers of inflammation among healthy subjects. Overall, our results revealed that long-term ET induced significant decrease in IL-6, CRP, and TNFα levels. Long-term ET-induced reduction of IL-6 was more evident in participants with a BMI >25, engaged in exercise for less than 12 weeks and engaged in type of multicomponent exercise. On the other hand, Long-term ET-induced decrease in CRP levels was associated with participants involved in exercise for more than 12 weeks and involved in aerobic exercise. Long-term ET-induced decrease in TNFα levels was associated with participants of younger than 60 years of age, involved in exercise for more than 12 weeks, and involved in exercise of moderate intensity.

Elevated levels of circulating inflammatory markers such as IL-6, TNFα and CRP, induces the development of chronic low-grade inflammation which has been identified as a risk predictor for several diseases such as T2DM (Pradhan et al., 2001) and dementia (Leonard, 2007). Our findings showed that engaging in ET for over 12 weeks effectively reduced the levels of CRP and TNFα. This is consistent with previous studies which showed that ET interventions conducted over longer durations can minimize inflammation. A meta-analysis based on elderly participants showed that resistance training alone can reduce CRP and TNFα when conducted for more than 12 weeks (Sardeli et al., 2018). It is well known that one-time exercise interferes with cell homeostasis leading to inflammation, while repeated exercise training improves immunocompetence (Scheffer and Latini, 2020). Skeletal muscle is an endocrine organ. During muscle contraction, it can produce cytokines and release them into the blood, which can systematically reduce inflammation (Furman et al., 2019). It was reported that several myokines, such as CRP, peaked at the end of exercise and returned to baseline levels within several hours (Keller et al., 2005; Fischer, 2006), which could mediate metabolic changes followed by increase anti-inflammatory cytokine (Febbraio and Pedersen, 2002), thus activate anti-inflammatory immune response. Therefore, long-term exercise may contribute to lower basal levels of inflammatory biomarkers.

Consistent with the previous studies (Scheffer and Latini, 2020), we also found that exercise of moderate intensity was more beneficial for the reduction of CRP and TNFα levels. The intensity of training has been shown to affect inflammatory markers in a dose-dependent manner (Fatouros et al., 2009). Several studies have shown that ET of moderate intensity promotes anti-inflammatory response, while ET of high intensity promotes an inflammatory response (Pinho et al., 2010; Lindsay et al., 2015). However, a recent meta-analysis based on 27 studies, including 17 studies with patients, revealed that intensity of exercise did not influence the chronic inflammatory response (Rose et al., 2021). Nevertheless, the study by Rose et al. only included studies that compared more than two different intensities of exercise, with significant heterogeneity in the exercise prescription among the studies. As a result, total exercise volume may have confounded the outcomes. Overall, our meta-analysis suggested that long-term three or more times a week of moderate intensity ET could significantly reduce the level of inflammatory biomarkers in healthy adults, and that such exercise frequency and intensity were consistent with the guidelines of the American College of Sports Medicine (Garber et al., 2011).

Aging is inherently associated with chronic increase in cytokine concentration, a condition termed as inflammaging (Franceschi and Campisi, 2014). Indeed, a meta-analysis from observational studies demonstrated that the frailty and pre-frailty in the elderly were associated with higher inflammatory parameters, especially CRP and IL-6 (Soysal et al., 2016). Therefore, the elderly are more likely to benefit from the anti-inflammatory effect of ET. Meanwhile, further studies are needed to establish the relationship between age and the anti-inflammatory effect of PE, as well as the underlying mechanisms.

There are several limitations in this study. First, there was no blinding in 34 of the 38 included studies, which may have introduced some bias. The high heterogeneity observed across studies might lead to degradation of the credibility of the results. Thus, there is need to excess caution when interpreting the results of our study. The high heterogeneity may have been due to the use of different experimental designs, particularly study samples, interventions and measures of outcome, among the different studies. Although we explored the source of heterogeneity via subgroup analysis and meta-regression, some of the results were also limited by the differences in sample size between subgroups. Furthermore, some of our results from subgroup analysis showed publication bias, which may affect the validity of the effects observed. However, most of these results remained unchanged or showed greater effect size after adjustment using the trim and fill method, thus making the results credible.

## Conclusion

Taken together, long-term ET induced significant decrease in IL-6, CRP, and TNFα levels in healthy subjects compared to non-active interventions. Long-term ET of moderate intensity and conducted for more than 12 weeks induced more pronounced anti-inflammatory effects in healthy subjects. There is need for future RCTs to explore the optimum long-term ET protocol beneficial for people of different ages.

## Data availability statement

The original contributions presented in the study are included in the article/Supplementary material, further inquiries can be directed to the corresponding authors.

## Author contributions

Y-HW: Data curation, Visualization, Writing – original draft. JT: Data curation, Visualization, Writing – original draft. H-HZ: Data curation, Validation, Visualization, Writing – original draft. MC: Data curation, Visualization, Writing – review & editing. YZ: Data curation, Visualization, Writing – review & editing.

## Funding

This research was funded by the Science and Technology Project of Education Department of Jiangxi Province (GJJ2203217), and the Science and Technology Innovation Project of General Administration of Sport of China (22KJCX035).

## Conflict of interest

The authors declare that the research was conducted in the absence of any commercial or financial relationships that could be construed as a potential conflict of interest.

## Publisher’s note

All claims expressed in this article are solely those of the authors and do not necessarily represent those of their affiliated organizations, or those of the publisher, the editors and the reviewers. Any product that may be evaluated in this article, or claim that may be made by its manufacturer, is not guaranteed or endorsed by the publisher.
