# Supplementary material for: Long-term exercise training and inflammatory biomarkers in healthy subjects: a meta-analysis of randomized controlled trials
Source: Front Psychol. 2023 Aug 30;14:1253329. doi: 10.3389/fpsyg.2023.1253329 (PMC10499556; doi:10.3389/fpsyg.2023.1253329)
Supplement: Supplementary file 2 [file Table_2.DOCX]

**Supplementary table 1**

Example search strategy for the Pubmed database.

**((randomized controlled trial [Publication Type])OR (randomized[Title/Abstract]) OR (placebo[Title/Abstract])) AND ((Inflammation[Mesh]) OR (Interleukins[Mesh]) OR (Interleukin[Title/Abstract]) OR (Tumor Necrosis Factors[Mesh]) OR (Necrosis Factors, Tumor[Title/Abstract]) OR (TNF Receptor Ligands[Title/Abstract]) OR (Receptor Ligands, TNF[Title/Abstract]) OR (Tumor Necrosis Factor Superfamily Ligands[Title/Abstract]) OR (Cytokines[Mesh]))AND ((Exercise[Mesh]) OR (Exercise training[Title/Abstract]) OR (Long term exercise training [Title/Abstract]) OR (Exercises, Physical[Title/Abstract]) OR (Physical Exercise[Title/Abstract]) OR (Physical Exercises[Title/Abstract]) OR (Exercise, Isometric[Title/Abstract]) OR (Exercises, Isometric[Title/Abstract]) OR (Isometric Exercises[Title/Abstract]) OR (Isometric Exercise[Title/Abstract]) OR (Exercise, Aerobic[Title/Abstract]) OR (Aerobic Exercises[Title/Abstract]) OR (Exercises, Aerobic[Title/Abstract]) OR (Aerobic Exercise[Title/Abstract]) OR (Exercise Therapy[Mesh]) OR (Therapy, Exercise[Title/Abstract]) OR (Exercise Therapies[Title/Abstract]) OR (Therapies, Exercise[Title/Abstract]) OR (Exercise Movement Techniques[Mesh]) OR (Movement Techniques, Exercise[Title/Abstract]) OR (Exercise Movement Technics[Title/Abstract]) OR (Pilates-Based Exercises[Title/Abstract]) OR (Exercises, Pilates-Based[Title/Abstract]) OR (Pilates Based Exercises[Title/Abstract]) OR (Pilates Training[Title/Abstract]) OR (Training, Pilates[Title/Abstract]))**

Example search strategy for other databases.

**((****"randomized controlled trial" OR randomized OR placebo) AND (****"Inflammation" OR "Interleukins" OR "Interleukin" OR "Tumor Necrosis Factors" OR "TNF Receptor Ligands" OR "Tumor Necrosis Factor Superfamily Ligands" OR "Cytokines") AND ("Exercise" OR "Physical Exercise" OR " Exercise training " OR " Long term exercise training " OR "Pilates-Based Exercises"))**

**Supplementary table2 Grades of Recommendation, Assessment, Development and Evaluation (GRADE) quality of evidence**

| **Outcome** | **Risk of**  **bias** | **Inconsistency** | **Indirectness** | **Imprecision** | **Publication**  **bias** | **Effect**  **size** | **Plausible**  **residual**  **confounding** | **Dose-response**  **gradient** | **GRADE**  **rating** |
| --- | --- | --- | --- | --- | --- | --- | --- | --- | --- |
| IL-6 | 0 | -1^a^ | 0 | 0 | 0 | 0 | 0 | 0 | Moderate |
| CRP | 0 | -1^a^ | 0 | 0 | 0 | 0 | 0 | 0 | Moderate |
| TNFα | 0 | -1^a^ | 0 | 0 | -1^c^ | 0 | 0 | 0 | Low |

^a.^ Significant and unexplained variability exists in the primary analysis; ^b^. Effect size overlaps 0 in the primary analysis; ^c^, p < 0.1 on Begg’s or Egger’s regression test.

Abbreviations: CRP, C-reactive protein; IL-6, Interleukin 6; TNFα, tumour necrosis factor alpha.


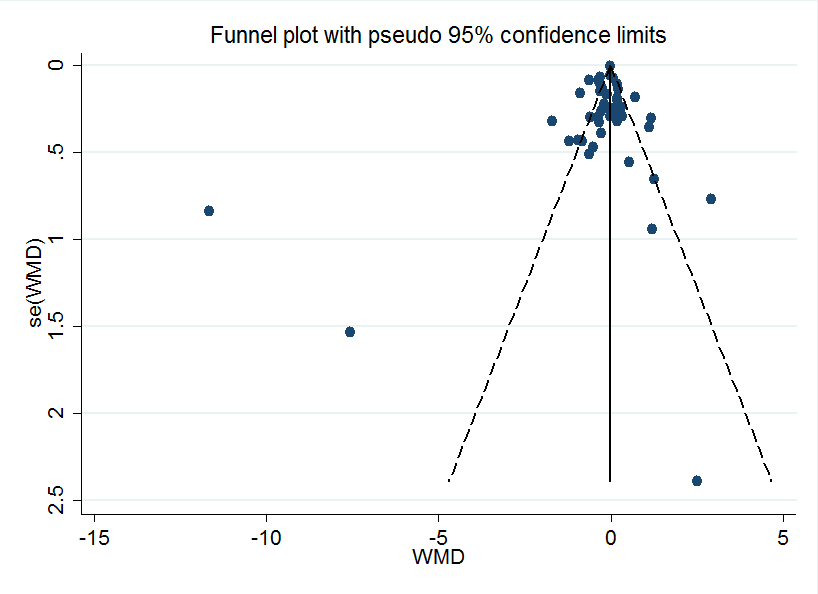


**FIGURE S1. The funnel plot for interleukin-6**

**
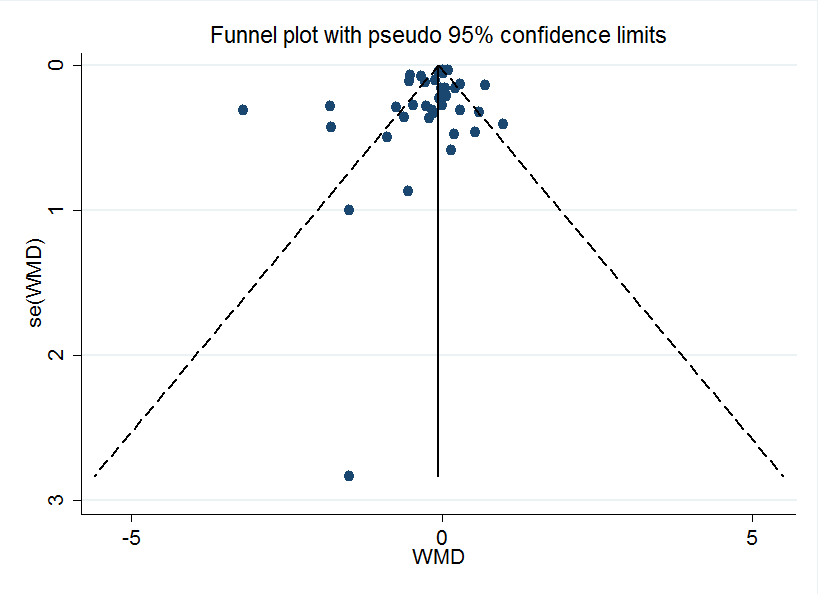
**

**FIGURE S2. The funnel plot for C-reactive protein**

**
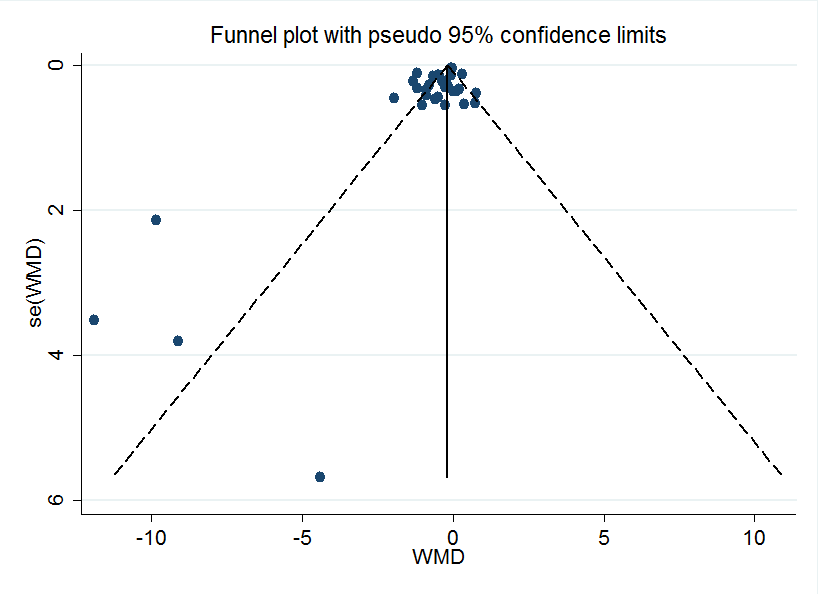
**

**FIGURE S3. The funnel plot for tumor necrosis factor alpha**
